# Spatio-temporal evolution and driving factors of carbon storage in the Western Sichuan Plateau

**DOI:** 10.1038/s41598-022-12175-8

**Published:** 2022-05-17

**Authors:** Mingshun Xiang, Chunjian Wang, Yuxiang Tan, Jin Yang, Linsen Duan, Yanni Fang, Wenheng Li, Yang Shu, Mengli Liu

**Affiliations:** 1 College of Tourism and Urban-Rural Planning, Chengdu University of Technology, Chengdu, 610059 China; 2 College of Earth Science, Chengdu University of Technology, Chengdu, 610059 China; 3 Research Center for Human Geography of Tibetan Plateau and its Eastern Slope (Chengdu University of Technology), Chengdu, 610059 China; 4 College of Management, Sichuan Agricultural University, Chengdu, 611130 China

**Keywords:** Climate sciences, Ecology, Environmental sciences, Environmental social sciences

## Abstract

The carbon sequestration function of the ecosystem is one of the most important functions of ecosystem service, and it of great significance to study the spatio-temporal differentiation of carbon storage for promoting regional sustainable development. Ecosystems on the Western Sichuan Plateau are highly variable, but its spatio-temporal differentiation and driving factors are not yet clear. In this study, on the basis of land use monitoring data, meteorological and demographic data interpreted from Landsat remote sensing image, and through GIS analysis tools, the carbon storage module of InVEST (Integrated Valuation of Ecosystem Services and Trade-offs) model was used to estimate carbon storage and geodetector was used to detect the driving factors of carbon storage spatial differentiation. The results show that: (1) The carbon storage increased to 1.2455 × 10^10^ t from 1.2438 × 10^10^ t in the past 20 years, the ecosystem developed in a healthy way overall. (2) Carbon storage show High-High and Low-Low aggregation characteristics, but the area decreased by 1481.81 km^2^ and 311.11 km^2^ respectively, and the spatial cluster effect gradually weakened. (3) HAI is the leading factor causing the spatio-temporal differentiation of regional carbon storage, followed by temperature and NDVI; the interaction between factors significantly enhances the spatial differentiation of carbon storage, indicating that the change of carbon storage is the result of the joint action of natural and socioeconomic factors. The results of the study provide some theoretical basis for the development of differentiated ecological regulation models and strategies, and help to promote high-quality regional development.

## Introduction

The trend of global warming is becoming more and more obvious, which has attracted the attention of people from all walks of life around the world^1,2^. The IPCC AR6 Working Group 1 report states that "climate change will be aggravated in all regions in the coming decades unless greenhouse gas emissions are reduced immediately and on a large scale. As one of the most important functions of ecosystem service, the carbon sequestration function of ecosystem plays a major role in global carbon cycle, atmospheric CO_2_ absorption and climate change^3,4^. In pursuing economic growth, the solid function of ecosystem carbon sequestration which is kept improving represents an important way to enhance GDP increase^5^. There is a response relationship between carbon storage and Land Use and Cover Changes (LUCC). LUCC affects the carbon storage changes in the whole area by influencing the carbon storage of vegetation and soil in the ecosystem, and land use changes are usually accompanied by significant carbon exchange^6,7^. However, research also indicates that LUCC of terrestrial ecosystems has become an important source of carbon emissions, which is second only to the burning of fossil fuel^8,9^. Research on changes in carbon storage based on LUCC has become one of the main methods for monitoring ecosystem service function^10,11^.

The biomass method, stock volume method, chamber method, and sampling method have relatively high accuracy in estimating carbon storage^12^, but it is difficult to reflect carbon storage changes over long time series and large scales^13^. The InVEST model provides new technology for conducting spatial expression, dynamic analysis, and quantitative evaluation of ecosystem service function^14^. More importantly, the InVEST model can easily be used to assess the impact of climate and LUCC changes on ecosystem carbon storage^11^. Changes in LUCC alter the structure (biomass, species composition) and function (energy balance, carbon cycle, biodiversity) of the ecosystem. The Carbon module of InVEST Model takes land use data as the main data source and is suitable for large-scale and long-term carbon storage estimation, so it is widely used in carbon cycle assessment and simulation of terrestrial ecosystems at different scales^15–17^. For example, Clerici et al. used the InVEST model to assess the impact of LUCC and climate change on carbon sequestration services in the Andes Mountains of Colombia^18^. Based on this model, He et al. evaluated the impact of urban expansion on regional carbon emissions and believed that the model is suitable for evaluating the impact of urban expansion on ecology after verification^19^. Li et al. evaluated the carbon storage change in the Loess Plateau and believed that carbon density has a strong spatial correlation with NDVI^2^. There are also studies combining CA-Markov, CLUE-S and other models to predict the future change trend of carbon storage^13,20^. In general, the research results of regional carbon storage assessment based on InVEST model are abundant, which provides an important basis for dynamic monitoring and evaluation of regional ecosystem service function.

With complex geographical environment, large altitude differences and obvious climate change, the Western Sichuan Plateau, located at the southeast edge of the Qinghai-Tibet Plateau, is a typical ecologically fragile area and ecoclimate sensitive area in China and an important ecological barrier and water conservation area in the upper reaches of the Yangtze River and Yellow River^21,22^. The rich vegetation types, high altitude, low temperature and slow decomposition rate of soil organic matter in the Western Sichuan Plateau making it one of the areas with the highest carbon density in China^23^. Meanwhile, the peat organic carbon in the Western Sichuan Plateau accounts for more than 80% of Sichuan Province, which is one of the main distribution areas of peat organic carbon in China and an important carbon sink and storage area of terrestrial ecosystems in China^24,25^. With the global climate change and the continuous growth of population, the land development and utilization activities in the Western Sichuan Plateau continue to intensify; coupled with strong tectonic movement in the area and frequent occurrence of geological disasters such as landslide and debris flow, the ecological environment is facing great challenges, especially on the carbon cycle and carbon balance^26,27^.

Currently, the research results on carbon storage estimation are relatively common, but the research on the spatial differentiation of carbon storage and its driving factors on the spatio-temporal scale is rare, especially related research results for plateau areas are extremely lacking. This paper takes the Western Sichuan Plateau as the research object, and uses the InVEST model and geodetector as the main research methods. The main purposes of this study are: (1) to find out the carbon storage and its spatio-temporal pattern of the Western Sichuan Plateau; (2) to explore the driving factors of carbon storage change in the study area; (3) to study the relationship between carbon storage changes and dominant factors. This study has contributed to the improvement of cognition of the ecosystem functions in the Western Sichuan Plateau, and is of great significance to the ecological barrier construction and regional ecological security in the Western Sichuan Plateau and the entire Qinghai-Tibet Plateau.

## Materials and methods

### Study area

**Figure 1:**
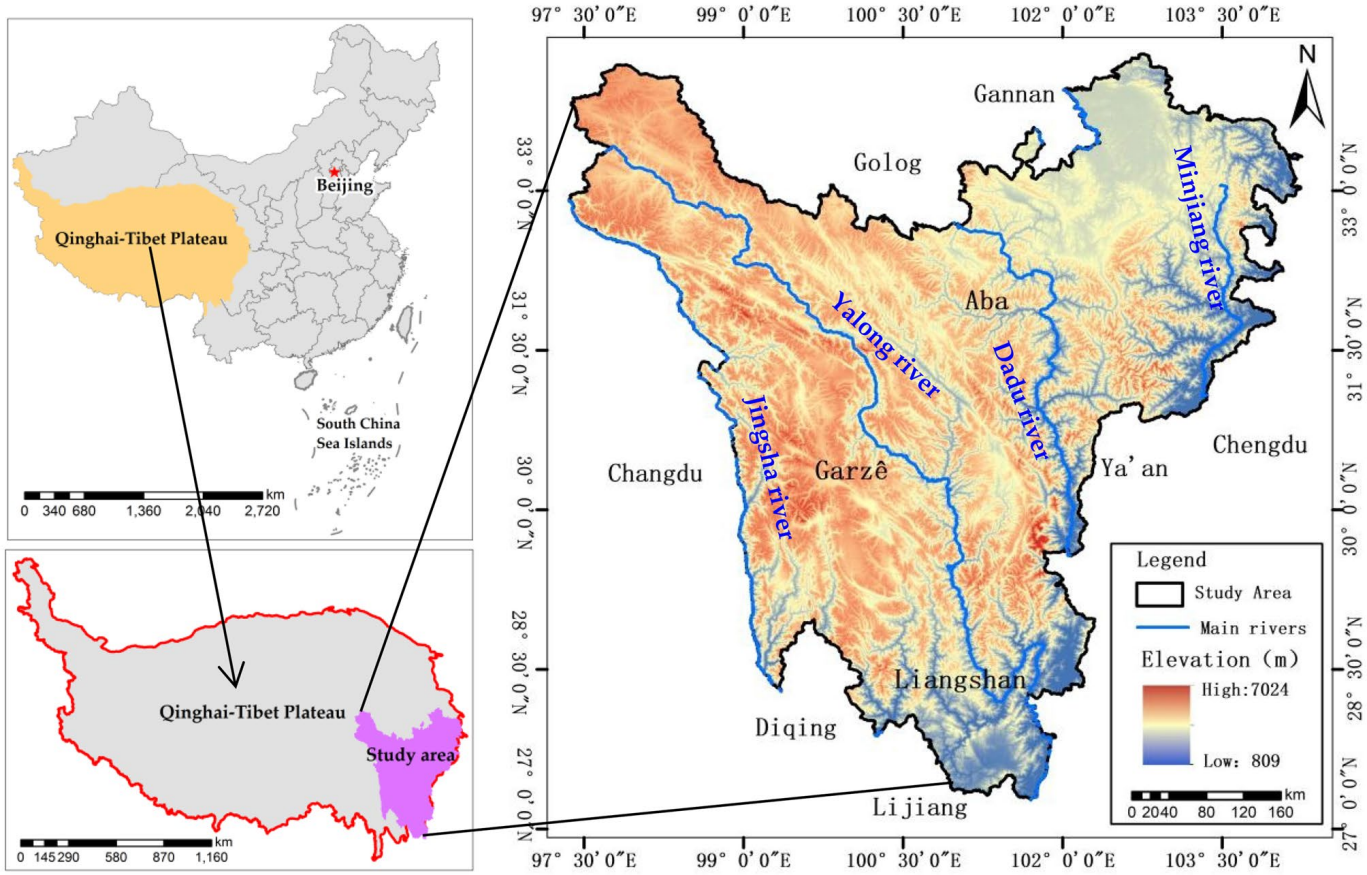
Location of the study area. The map is created in the support of ArcGIS 10.2 (ESRI). The China map and Western Sichuan Plateau boundary data were collected from Resources and Environmental Science and Data Center (http://www.resdc.cn/). The Qinghai-Tibetan Plateau boundary data were collected from the Global Change Research Data Publishing & Repository (http://www.geodoi.ac.cn/WebCn/Default.aspx).

With an area of about 2.33 × 10^5^ km^2^, the Western Sichuan Plateau (27.11°–34.31°N and 97.36°–104.62°E) is located in the transition zone between the Qinghai-Tibet Plateau and the Sichuan Basin, including all of Garze Prefecture and Aba Prefecture, and parts of Liangshan Yi Autonomous Prefecture^28^ (Fig. 1). With an altitude of 780–7556 m, this area is dominated by mountain and ravine areas and high mountain and plateau areas, and the terrain is high in the west and low in the east. The climate belongs to the subtropical plateau monsoon climate, with large temperature difference between day and night and abundant sunshine. The annual average temperature is about 9.01–10.5 °C, and the precipitation is about 556.8–730 mm^28^. The study area is rich in water resources, including the Yalong River, Minjiang River and other important river systems in the upper reaches of the Yangtze River, and the Baihe River, Heihe river and other river systems of the Yellow River. The main types of soil are plateau meadow soil dark brown soil, brown soil, cold frozen soil and cinnamon soil, and the main vegetation types are alpine meadow and scrub. With rich and diverse soil vegetation types and distinctive vertical zonal distribution characteristics, it is one of the global biodiversity conservation hotspots^29^.

### Data source and processing

Multisource archival data were used in this study (Table 1). The land use remote sensing monitoring data, administrative boundary data and geological disaster vector data were obtained from Resources and Environmental Science and Data Center. The spatial resolution of land use remote sensing monitoring data is 30 × 30 m, including 6 first-level classification and 26s-level classification. The first-level classification includes cropland, woodland, grassland, water body, built-up land, and unused land. The accuracy of remote sensing classification is not less than 95% for cropland and built-up land, not less than 90% for grassland, woodland, and water body, and not less than 85% for unused land, which meets the need of the research. Landsat remote sensing monitoring data is used as the main information resources, among which Landsat-TM/ETM remote sensing monitoring data is used in 2000, 2005, 2010 and Landsat 8 remote sensing monitoring data is used in 2015 and 2020. In light of actual conditions and the implementation of policies and philosophies including the natural forest protection project, return of farmland to forest, land remediation, ecological civilization, the period from 2000 to 2020 is selected as the study period, and the land use data of each period is cropped using ArcGIS 10.2 to reclassify the 26 secondary classifications into cropland, woodland, grassland, water body, built-up land and unused land.

**Table 1:** Characteristics of data used for the study.

Data	Type	Resolution/scale	Year	Data source
Land use	Raster	30 m	2000, 2005, 2010, 2015, 2020	http://www.resdc.cn/
Landsat	Raster	Landsat-TM/ETM 30 m Landsat 8 15 m	2000, 2005, 2010, 2015, 2020	https://earthexplorer.usgs.gov/
SRTM DEM	Raster	20 m (horizontal) 16 m (elevation accuracy) 30 m (spatial resolution)	2000	http://www.resdc.cn/
Qinghai-Tibetan Plateau boundary	Vector	–	2014	http://www.geodoi.ac.cn/WebCn/Default.aspx
GDP	Raster	1000 m	2000, 2005, 2010, 2015, 2020	http://www.resdc.cn/
NDVI	Raster	1000 m	2000, 2005, 2010, 2015, 2020	http://www.resdc.cn/
Population	Raster	1000 m	2000, 2005, 2010, 2015, 2020	http://www.resdc.cn/
Temperature	Raster	1000 m	2000, 2005, 2010, 2015, 2020	http://www.resdc.cn/
Rainfall	Raster	1000 m	2000, 2005, 2010, 2015, 2020	http://www.resdc.cn/

The DEM data were obtained from SRTM (Shuttle Radar Topography Mission) of Resources and Environmental Science and Data Center, the spatial resolution of 30 × 30 m, absolute horizontal accuracy ± 20 m, absolute elevation accuracy ± 16 m, elevation and slope are extracted from the downloaded DEM. The Qinghai-Tibetan Plateau boundary data were collected from the Global Change Research Data Publishing & Repository. Data of carbon density of different land types were obtained from Chinese Ecosystem Research Network Data Center (http://www.nesdc.org.cn/).

A total of 29,284 evaluation units were collected for spatial grid processing of the Western Sichuan Plateau according to 3 km × 3 km by ArcGIS 10.2. The impact factors obtained in this study include grid data per kilometer of GDP spatial distribution, grid data per kilometer of population spatial distribution, annual mean temperature spatial interpolation data, annual mean rainfall spatial interpolation data, long-term normalized difference vegetation index (NDVI) comes from Resources and Environmental Science and Data Center with a resolution of 1 km × 1 km. The Human Active Index (HAI), with a resolution of 30 m × 30 m, can be calculated by formula^30,31^, and the factors are discretized into the data type required for the geodetector by the natural breakpoint method.

### Methods

#### The InVEST model

The InVEST model was developed by Stanford University, the University of Minnesota, the Nature Conservancy and the World Wide Fund for Nature (WWF). The model's terrestrial ecosystem services assessment includes four modules: soil conservation, water retention, carbon storage and biodiversity assessment, and provides an overall measurement of regional ecosystem services^32^. The carbon storage model of the InVEST model divides the carbon storage of the ecosystem into 4 basic carbon pools, namely above-ground carbon, underground carbon, soil carbon, dead organic matter carbon^7^.

The calculation formula of total carbon storage in the Western Sichuan Plateau is as follows^7^:

1 $$C_{total} = C_{above} + C_{below} + C_{soil} + C_{dead}$$

In formula (1), *C*_*total*_ is the total carbon storage; *C*_*above*_ is the above-ground carbon storage; *C*_*below*_ is the underground carbon storage; *C*_*soil*_ is the soil carbon storage, and *C*_*dead*_ is the dead organic matter carbon storage.

Based on the carbon density and land use data of different land use type, the carbon storage of each land use type in the Western Sichuan Plateau is calculated by the formula^7^:

2 $$C_{{\text{total}}i} = (C_{{\text{above}}i} + C_{{\text{below}}i} + C_{{\text{soil}}i} + C_{{\text{dead}}i}) \times A_{i}$$

In formula (2), *i* is the average carbon density of each land use, and *A*_*i*_ is the area of this land used.

The carbon density data of different land use types in this study were obtained from the shared date of the National Ecological Science Data Center and some documents^33–37^. Since the carbon density data were collected from the results of studies in different parts of China, the selected documents should be close to or similar to the study area as far as possible to avoid excessive data gap. At the same time, the carbon density varies with climate, soil properties and land use^38^, so the carbon density should be modified according to the climate characteristics and land use types of the Western Sichuan Plateau. Existing research results show that the carbon density is positively correlated with annual precipitation and weakly correlated with annual average temperature. The quantitative expression of the relationship between carbon density and temperature and precipitation is as follows^39–42^:

3 $$C_{SP} = 3.3968 \times P + 3996.1\;\;\left( {{\text{R}}^{{2}} = 0.{11}} \right)$$

4 $$C_{BP} = 6.7981e^{0.00541p}\;\;\;\left( {{\text{R}}^{{2}} = 0.{7}0} \right)$$

5 $$C_{BT} = 28 \times {\text{T}} + 398\;\;\left( {{\text{R}}^{{2}} = 0.{47,}\;{\text{P}} < 0.0{1}} \right)$$

In these formula, *C*_*SP*_ is the soil carbon density (kg m^−2^) based on the annual precipitation; *C*_*BP*_ is the biomass carbon density (kg m^−2^) based on the annual precipitation; *C*_*BT*_ is the biomass carbon density (kg m^−2^) based on annual average temperature; *P* is the average annual precipitation (mm), and *T* is the annual average temperature (°C). According to the data of China Meteorological Data Service Centre (http://data.cma.cn/), in the past 20 years, the average annual temperature of China and the Western Sichuan Plateau was 9.0 °C and 6.3 °C, and the average annual precipitation was 643.50 mm and 812.65 mm respectively.

The modified formula of carbon density in the Western Sichuan Plateau is as follows^7^:

6 $$K_{BP} = \frac{C^{\prime}{_{BP}}}{{C^{\prime\prime}{_{BP}}}}$$

7 $$K_{BT} = \frac{C^{\prime}{_{BT}}}{{C^{\prime\prime}{_{BT}}}}$$

8 $$C_{BT} = 28 \times T + 398\;\;\left( {{\text{R}}^{{2}} = 0.{47,}\;{\text{P}} < 0.0{1}} \right)$$

9 $$K_{S} = \frac{C^{\prime}{_{SP}}}{{C^{\prime\prime}{_{SP}}}}$$

In these formula, *K*_*BP*_ is the modified indices of precipitation factor in biomass carbon density; *K*_*BT*_ is the modified indices of temperature factor; *C'*_*BP*_ and *C''*_*BP*_ are the biomass carbon density obtained from annual precipitation in the Western Sichuan Plateau and the whole country respectively. *C'*_*BT*_ and *C''*_*BT*_ are the biomass carbon density obtained from annual average temperature; *C'*_*SP*_ and *C''*_*SP*_ are the soil carbon density data obtained from annual average temperature; *K*_*B*_ and *K*_*S*_ are the biomass carbon density modified indices and soil carbon density modified indices respectively. The carbon density values of each land use type after modified in the Western Sichuan Plateau are shown in Table 2.

**Table 2:** Carbon density values of different land use types in the Western Sichuan plateau (t hm^−2^).

Land use type	*C*_*above*_	*C*_*below*_	*C*_*soil*_	*C*_*dead*_
Cropland	1.241	17.574	11.847	2.138
Woodland	9.233	33.670	17.355	3.073
Grassland	7.687	25.130	10.918	1.585
Water body	0.653	0.000	0.000	0.000
Built-up land	0.544	7.990	0.000	0.000
Unused land	0.283	0.000	2.361	0.000

#### Exploratory spatial analysis method

##### Global spatial autocorrelation

Global Moran’s I was used to describe the spatial differentiation characteristics of carbon storage in the study area, and the expression formula is as follows^43^:

10 $$I = \frac{{n\sum\nolimits_{i = 1}^{n} {\sum\nolimits_{j = 1}^{n} {w_{i,j} \left( {x_{i} - \overline{x} } \right)\left( {x_{j} - \overline{x} } \right)} } }}{{\sum\nolimits_{i = 1}^{n} {\sum\nolimits_{j = 1}^{n} {\omega_{ij} } } \sum\nolimits_{i = 1}^{n} {\left( {x_{i} - \overline{x} } \right)^{2} } }}$$

*w*_*ij*_ is the spatial weight; *x* is the attribute mean; *x*_*i*_ and *x*_*j*_ are the attribute values of elements *i*, *j*, respectively; *n* is the number of cells, and the correlation is considered significant when |Z| > 1.96.

##### Local indications of spatial association (LISA)

LISA reveals the local cluster characteristics of spatial unit attributes by analyzing the difference and significance between spatial units and surrounding units, and the expression formula is as follows^42^:

11 $$I_{i} (d) = \frac{{n(x_{i} - \overline{x} )\sum\nolimits_{j = 1}^{n} {w_{ij} (x_{j} - \overline{x} )} }}{{\sum\nolimits_{i = 1}^{n} {(x_{j} - \overline{x} )^{2} } }}$$

#### Correlation analysis

In order to evaluate the influence of natural factors and socioeconomic factors on carbon storage in the study area, the correlation coefficients of temperature, rainfall, NDVI, GDP, population density (PD), HAI and carbon storage were calculated according to the Pearson correlation coefficient method. The calculation formula is as follows^44^:

12 $$r_{xy} = \frac{{\sum\nolimits_{i = 1}^{n} {(M_{i} - \overline{x} )(y_{i} - \overline{y} )} }}{{\sqrt {\sum\nolimits_{i = 1}^{n} {(M_{i} - \overline{x} )^{2} \sum\nolimits_{i = 1}^{n} {(y_{i} - \overline{y} )} } } }}$$

*r*_*xy*_ represents the correlation coefficient between *x* and *y*; *M*_*i*_ represents the carbon storage in the *i*th year; y_i_ represents the value of the impact factor *Y* in the *i*th year, and ${\overline{\text{x}}}$ and ${\overline{\text{y}}}$ respectively represents the average value of carbon storage and impact factor in the research period over several years.

#### Human influence index analysis method

Land use is significantly spatially clustered in the study area^31^, and LUCC changes will have a certain impact on the structure and process of the ecosystem. HAI has the characteristics of spatial variability, which can reflect the impact of human activities on land use and landscape composition changes. In this study, Human Influence Index Analysis Method (HAI) index was used to analyze the correlation between carbon storage and human interference intensity in the Western Sichuan Plateau. The calculation formula is as follows^30^,

13 $$HAI = \sum\limits_{i = 1}^{n} {\left( {A_{i} P_{i} /TA} \right)}$$

*HAI* is Human Active Index; *A*_*i*_ is the total area of the *i*th land use type; *P*_*i*_ The intensity parameter of human impact reflected by type *i* land use type; *TA* is the total final surface area of land use type in evaluation unit; *n* is the number of land use types. Combined with the land use type of this study, *P*_*i*_ is assigned by Delphi method, in which cropland is 0.67, woodland is 0.13, grassland is 0.12, water body is 0.10, built-up land is 0.96, and unused land is 0.05^30,45^.

#### Geodetector

Geodetector is an algorithm that uses spatial heterogeneity principle to detect driving factors of carbon storage, which can quantitatively detect the influence of impact factors on carbon storage and explore the interaction between driving factors. Geodetector includes factor detection, risk detection, interaction detection and ecological detection^46^.

Differentiation and factor detection: the influence factors were discretized, and then the significance test of the difference in the mean values of the impact factors was conducted to detect the relative importance among the factors. The statistical quantity *q* is used to measure the explanatory power of impact factors on the carbon storage spatial differentiation and the value range of *q* is between 0 and 1. The larger the value, the stronger the explanatory power of the factor^47^.

14 $$q = 1{ - }\frac{{\sum\nolimits_{h = 1}^{L} {N_{h} \sigma_{h}^{2} } }}{{N\sigma^{2} }}$$

In this formula, *h* = 1, 2…, *L* is the classification or partition of variable (*Y*) or factor (*X*); *N*_*h*_ and N are layer *h* and regional number units respectively; and $\sigma_{h}^{2}$ and $\sigma_{{}}^{2}$ are the variance of the layer *h* and regional value *Y* respectively.

The variance of the regional value *Y* is calculated as follows,

15 $$\sigma^{2} = \frac{{\sum\nolimits_{i = 1}^{n} {(Y_{i} - \overline{Y} )^{2} } }}{N - 1}$$

where, *Y*_*i*_ and $\overline{Y}$ are the mean value of sample *j* and the region *Y*, respectively.

16 $$\sigma^{2} = \frac{{\sum\nolimits_{i = 1}^{{n_{h} }} {(Y_{h,i} - \overline{{Y_{h} }} )^{2} } }}{{N_{h} - 1}}$$

where, *Y* and $\overline{Y}$ are the value and mean value of sample *i* in layer *h*, respectively.

Interaction detection: it is used to identify the interaction between different impact factors *Xs*, that is, to evaluate whether the combined action of *X*_*1*_ and *X*_*2*_ will increase or weaken the explanatory power of vegetation coverage *Y*, or the influence of these factors on *Y* is independent of each other. The evaluation method is to first calculate the value *q* of the two factors *X*_*1*_ and *X*_*2*_ for *Y* respectively: *q(X*_*1*_*)* and *q(X*_*2*_*),* and calculate the value *q* of their interaction (the new polygon distribution formed by the tangent of the two layers of the superimposed variables *X*_*1*_ and *X*_*2*_) : *q*(*X*_*1*_ ∩ *X*_*2*_) and compare q(*X*_*1*_) and q(*X*_*2*_) with *q*(*X*_*1*_ ∩ *X*_*2*_)^46^.

## Results

### Spatio-temporal evolution of carbon storage

#### Characteristics of carbon storage from 2000 to 2020

Based on the InVEST model and the carbon density of different land use types in the Western Sichuan Plateau, the carbon storage from 2000 to 2020 was calculated and shown in Table 3. As can be seen from Table 3, the carbon storage of the Western Sichuan Plateau from 2000 to 2020 is 1.2438 × 10^10^ t, 1.2438 × 10^10^ t, 1.2465 × 10^10^ t, 1.2461 × 10^10^ t and 1.2455 × 10^10^ t, respectively. The overall change of carbon storage has changed much, showing the characteristics of increasing first and then slowly decreasing, but the overall trend is increasing.

**Table 3:** Carbon storage in the Western Sichuan Plateau from 2000 to 2020 (10^6^ t).

Year	Cropland	Woodland	Grassland	Water body	Built-up land	Unused land	Total
2000	197.27	5569.24	6623.99	0.79	1.19	45.66	12,438.15
2005	197.4	5569.63	6623.25	0.79	1.2	45.67	12,437.95
2010	196.81	5741.26	6478.07	1.01	1.95	45.91	12,465.02
2015	195.9	5738.58	6476.89	1.05	2.33	45.89	12,460.64
2020	195.39	5738.8	6471.07	1.13	2.75	45.82	12,454.95

From the perspective of the contribution rate of different land use types to the total carbon storage, the contribution rate of grassland is the highest from 2000 to 2020, accounting for 51.96% to 53.26%; followed by woodland, accounting for 44.78% to 46.08%; then comes cropland, contributing around 1.60%; the contribution rate of water body, construction land and unused land is low, accounting for less than 1%.

From the perspective of the variation rate of carbon storage, the total carbon storage increased by 0.14% from 2000 to 2020, with an average annual increase of 0.007%, showing no obvious change. Among them, the change of carbon storage is large from 2005 to 2010, increasing by 0.22%; the change rate of carbon storage in the two periods of 2010–2015 and 2015–2020 are close, decreasing by 0.04% and 0.05% respectively; and there is almost no change in carbon storage from 2000 to 2005.

**Figure 2:**
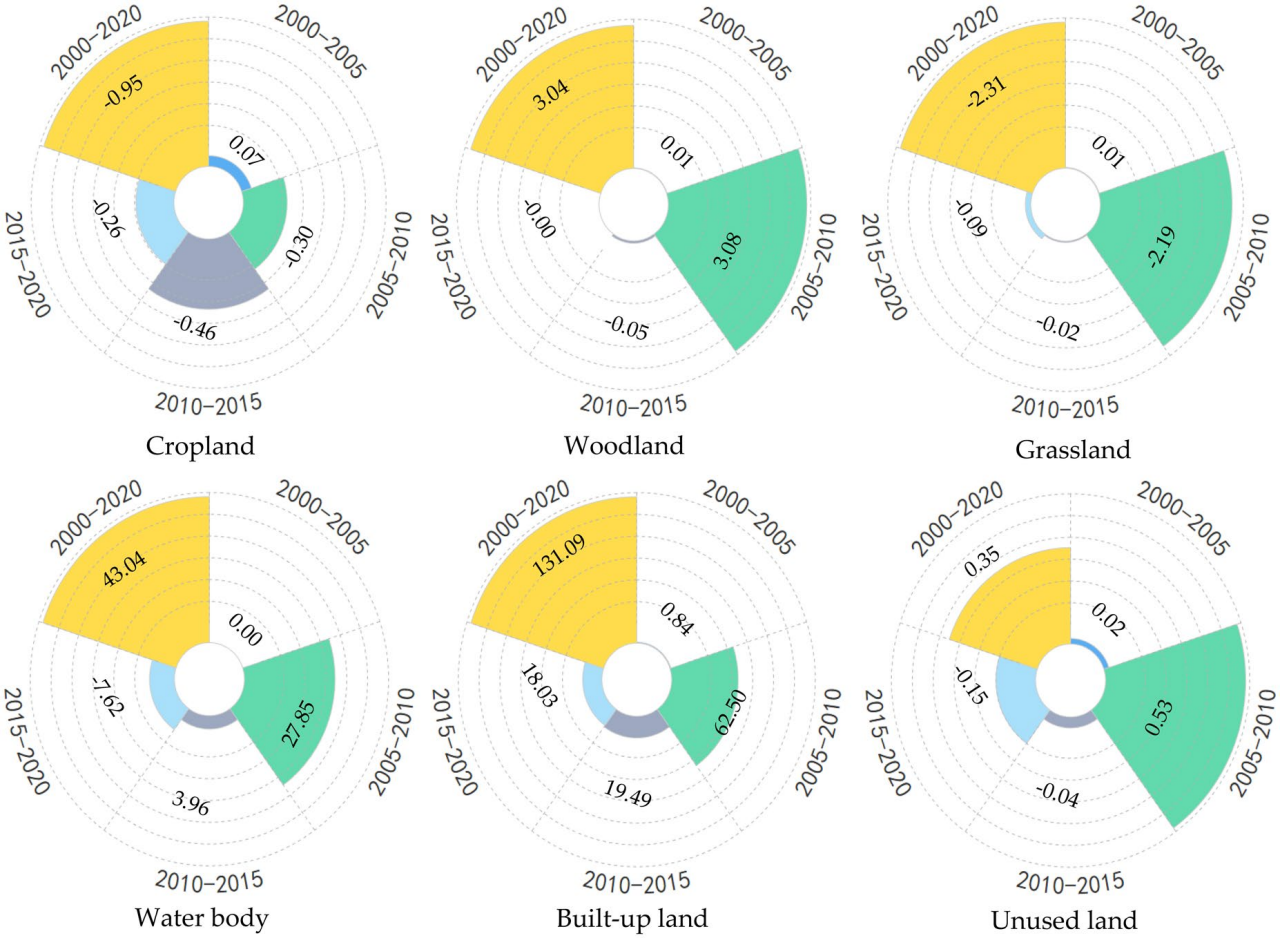
The change rate of carbon storage of different land use types in different periods (unit: %).

In terms of carbon storage change rate of different land use types (Fig. 2), the change rate from high to low are built-up land, water body, woodland, grassland, cropland and unused land, among which the carbon storage change rate of built-up land and water body far exceed those of other types of land, increasing by 131.09% and 43.04%, respectively. The period with the largest change in carbon storage on cropland was 2010–2015, with a decrease of 0.46%; the most significant change rate in carbon storage on woodland, grassland, water body, built-up land and unused land were all in 2005–2010; the smallest carbon storage change in each category were in 2000–2005.

#### Spatial variation characteristics of carbon storage

**Figure 3:**
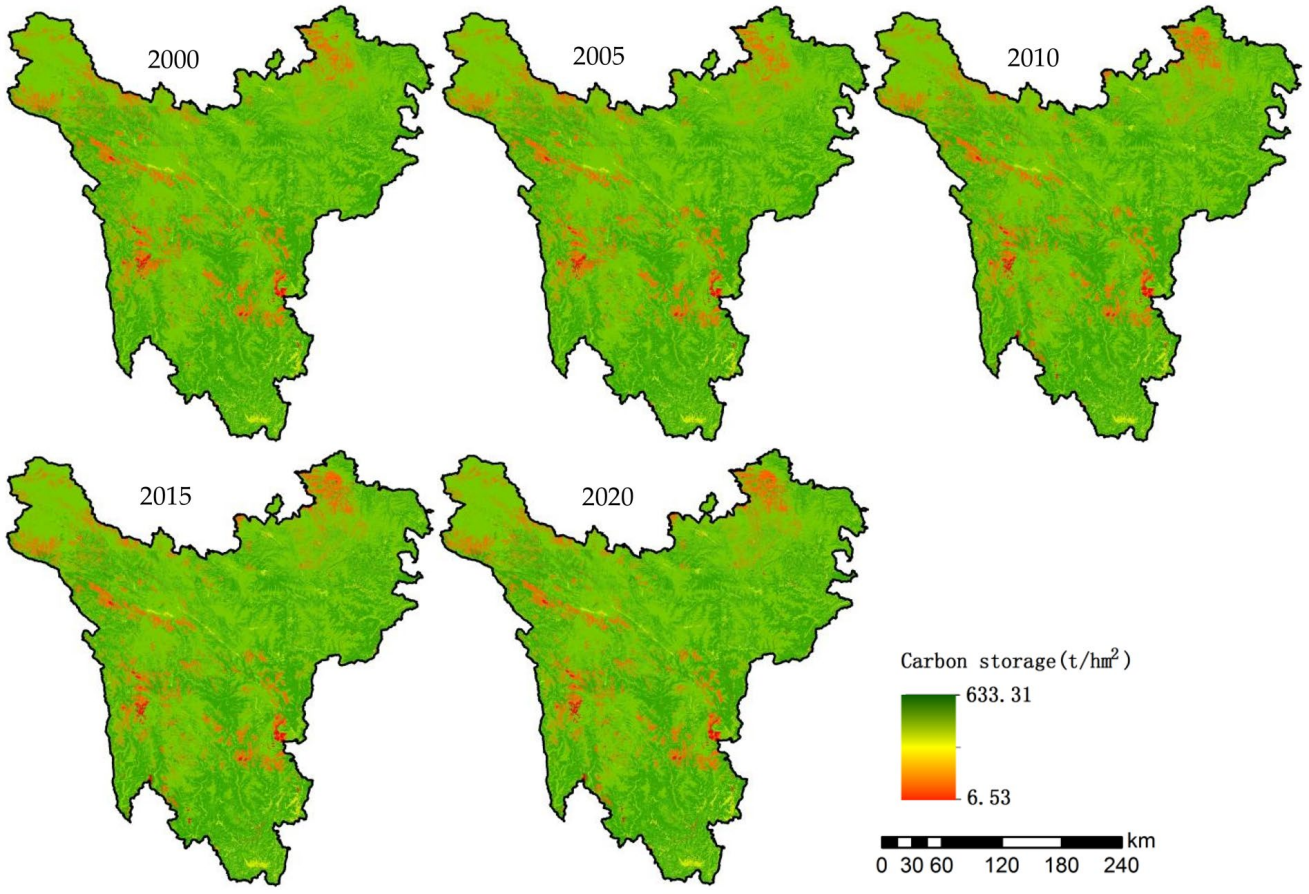
Spatio-temporal distribution of carbon storage from 2000 to 2020. Map generated with ArcGIS 10.2 (ESRI).

According to the spatial pattern of carbon storage in the Western Sichuan Plateau (Fig. 3), high carbon storage is distributed in the whole area, and the eastern and southern areas are higher than other areas as a whole. The carbon storage along the Yalong River, Dadu River and Minjiang River is also significantly higher, which is closely related to the high coverage rate of forest in these areas. Areas with low storage are scattered in Shiqu County and Dege County in the northwest, Ruoergai County and Batang County in the north, and Kangding County and Luding County in the middle, mainly because of the high proportion of unused land in these areas. The spatial distribution pattern of carbon storage is stable from 2000 to 2020. The area with no obvious change accounts for 94.27%; the area of reduced area is 11,436.00 km^2^, accounting for 4.42% of the total area, and the area of increased area is 3379.67 km^2^, accounting for 1.31%.

#### Spatial cluster characteristics of carbon storage

The carbon storage distribution data in the exploration area were gridded with a grid size of 3 km × 3 km, and Moran’s I index at the grid scale was calculated (Table 4). The Moran’s I value from 2000 to 2020 is greater than 0.7, p < 0.001, indicating that there is a significant spatial positive correlation and spatial cluster effect in the distribution of carbon storage in the study area. It also shows that the high value of carbon storage tends to accumulate and the low value tends to be adjacent, which has the characteristics of regional distribution. It should be noted that Moran’s I generally showed a slow decline trend from 2000 to 2020, indicating that the High-High Cluster and Low-Low Cluster effects of carbon storage in the study area tend to weaken gradually.

**Table 4:** Global Moran I of carbon storage from 2000 to 2020.

Year	Moran's *I*	z	p
2000	0.7331	174.86	0.0000
2005	0.7332	174.88	0.0000
2010	0.7260	173.16	0.0000
2015	0.7249	172.91	0.0000
2020	0.7243	172.77	0.0000

**Figure 4:**
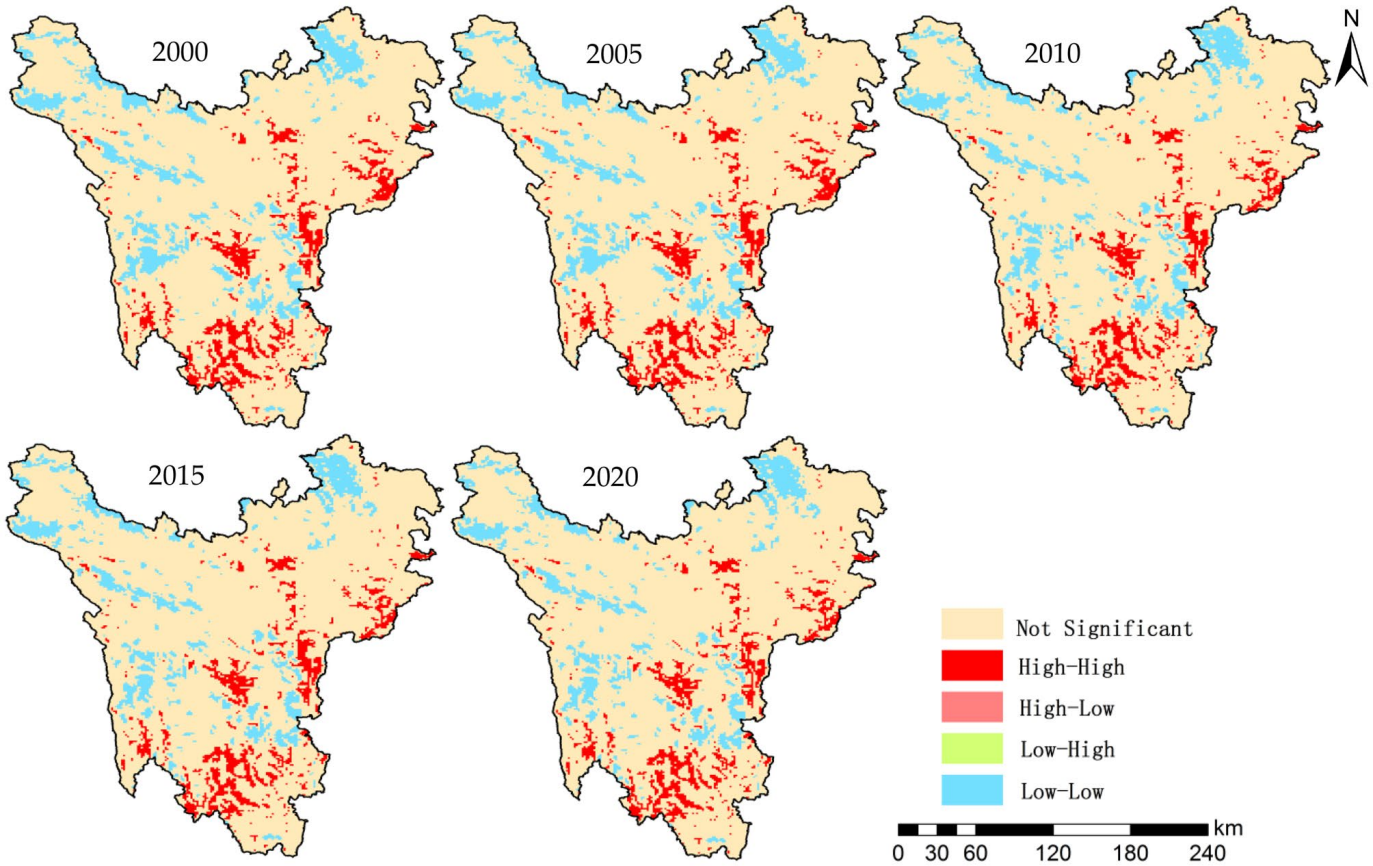
LISA cluster map of carbon storage in the Western Sichuan Plateau on a grid scale. Map generated with ArcGIS 10.2 (ESRI).

Using LISA to study the local cluster characteristics of spatial unit attributes of carbon storage, the spatial cluster is divided into four types: High-High Cluster, the carbon storage of the grid and its neighboring grid is high; High-Low Outlier, the carbon storage in the grid is high, but the carbon storage in the surrounding grid is low; Low-High Outlier, the carbon storage in the grid is low, but the surrounding neighborhood sgrid is high; Low-Low Cluster, the carbon storage of grid and surrounding neighborhood grid is low. The local spatial autocorrelation of carbon storage in the study area is shown in Fig. 4.

As shown in Fig. 4, the spatial cluster characteristics of the carbon storage in the Western Sichuan Plateau are obviously different. The two types, High-High Cluster and Low-Low Cluster, are highly significant, while High-Low Outlier and Low-High Outlier are not significant and the cluster characteristics show a weakening trend, which is consistent with the research results of Moran’s I. Specifically, the area of High-High Cluster accounted for 8–8.59%, mainly distributed in Li County, Wenchuan County in the east, Yajiang County, Kangding County in the middle, Muli County, Jiulong County in the south and other areas with high coverage of woodland. The largest change period is from 2005 to 2010 when the High-High Cluster area decreased by 1484.70 km^2^, followed by 2015–2020 when the area decreased by 144.25 km^2^; from 2000–2005 and 2010–2015, with area changes below 100 km^2^, the changes are not significant. There is little difference in the proportion of Low-Low Cluster area in different years, which is all around 10.7%; but it shows a decreasing trend, which mainly distributed in Shiqu County, Ganzi County and Dege County in the northwest, Ruoergai County and Hongyuan County in the northeast, Litang County and Batang County in the west and the junction of Luding County and Jiulong County in the east. Among them, the area changed the most is from 2005 to 2010, with a decrease of 240.81 km^2^; in other years, the change trend is relatively small, and the decrease area is less than 50 km^2^. High-Low Outlier and Low-High Outlier are distributed sporadically and spotty, accounting for about 0.01% of the total area.

### Correlation analysis of carbon storage change and driving factors

**Figure 5:**
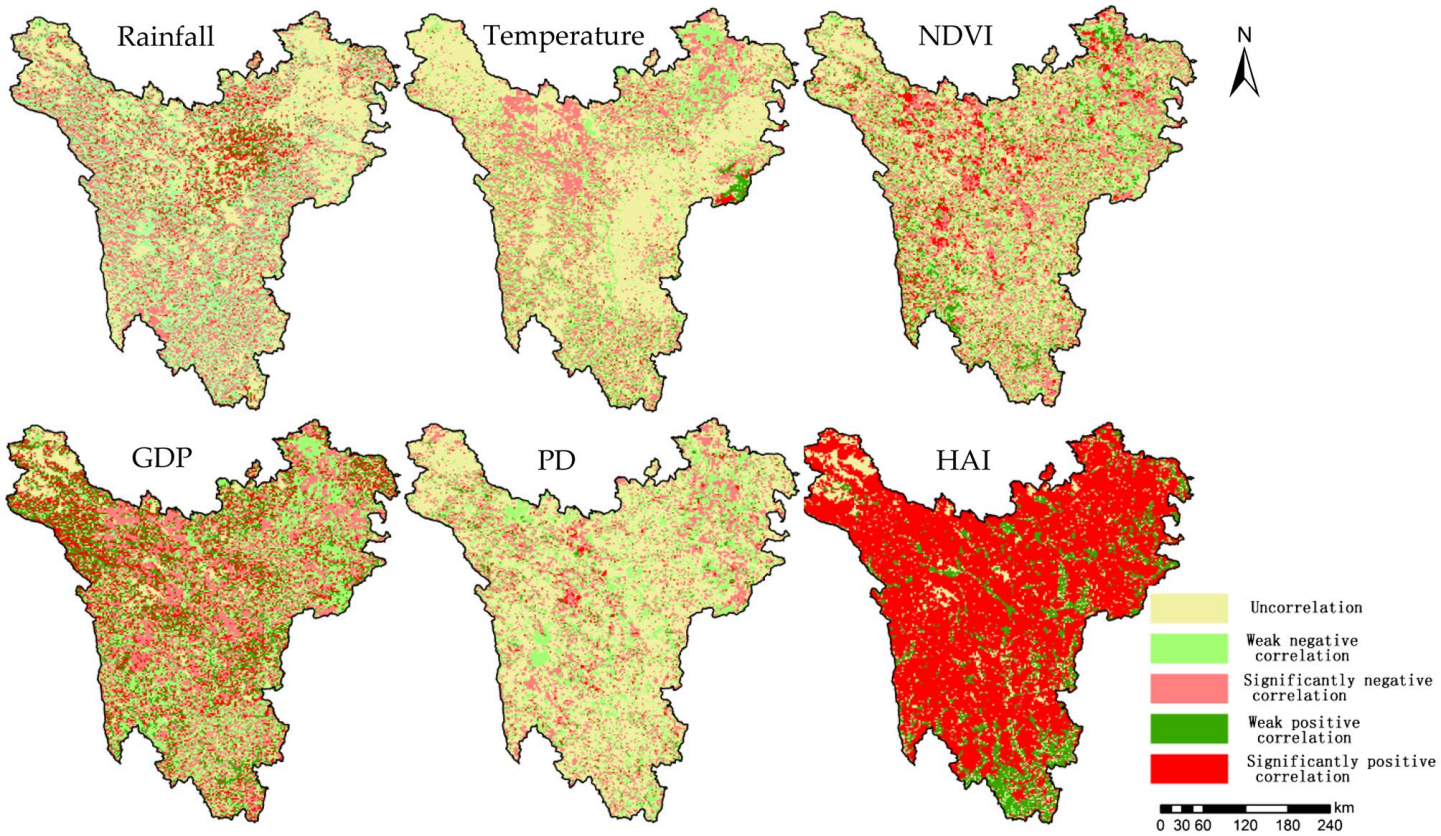
Correlation between carbon storage and impact factors in the study area. Map generated with ArcGIS 10.2 (ESRI).

In order to explore the relationship between the spatio-temporal evolution of carbon storage and the driving factors in the study area, the correlation coefficients between carbon storage and six factors such as rainfall and temperature were calculated pixel by pixel. The results are shown in Fig. 5.

It can be seen from Fig. 5 that HAI is the highest correlation factor with carbon storage change from 2000 to 2020, and the area with significant correlation account for 87.47% of the total area. Except for the relatively few Yanyuan County and Mianning County in the south, other counties account for a high proportion. Besides, the carbon storage is highly correlated with GDP, and the significantly correlated areas are also distributed throughout the whole area, with the overall characteristics of sparse in the west and dense in the east, high in the north and low in the south, and the uncorrelated areas only account for 10.3%. The effects of rainfall, NDVI and population density on the change of carbon storage are relatively close, with significant correlation between 14.10 and 18.09% and weak correlation about 55%; the areas with significant correlation between carbon storage and rainfall are mainly distributed in Kangding County, Rangtang County, Jinchuan County and Aba County. The areas with high correlation with NDVI are mainly distributed in high mountain and plateau areas such as Ruoergai County and Seda County. The high correlation with PD is mainly distributed with a spotty distribution and the spatial cluster characteristics are not obvious. The correlation between carbon storage change and temperature in the past 20 years is low, with only 4.27% of the areas significantly correlated and 53.02% uncorrelated. These results indicate that the carbon storage change in the Western Sichuan Plateau in recent 20 years is greatly affected by socioeconomic factors such as HAI, and relatively less affected by natural factors.

### Analysis on the driving factors of the spatial differentiation of carbon storage

#### Factor detection analysis based on geodetector

The spatial differentiation and changes of carbon storage in the study area are the result of the combined action of natural and socioeconomic factors. Geodetectors can be used to identify the contribution degree of various factors to the evolution of carbon storage and clarify the correlation among between various factors^46^. The driving forces of various impact factors on the spatial differentiation of carbon storage in the Western Sichuan Plateau are shown in Table 5.

**Table 5:** Factor detection for spatial heterogeneity of carbon storage.

Factors		q statistic	p value	Rank
Natural factors	Rainfall	0.0605	0	6
	Temperature	0.3027	0	2
	NDVI	0.2561	0	3
Socioeconomic factors	GDP	0.0981	0	4
	PD	0.0877	0	5
	HAI	0.5783	0	1

Table 5 shows that the driving forces of each impact factors on carbon storage change in the Western Sichuan Plateau are quite different. The overall order according to the size of q is HAI > Temperature > NDVI > GDP > PD > Rainfall. HAI is the leading factor of carbon storage spatial differentiation in the study area, accounting for 58.73%, indicating that human activities have a certain interference to the ecological environment of the Western Sichuan Plateau. The influences of temperature and NDVI are both greater than 25%, which are important factors influencing the spatial differentiation of carbon storage. The influence of GDP is more than 10%, which is a relatively important factor. However, population density and rainfall are both below 10% and have less driving force in the carbon storage spatial differentiation. The results show that natural factors and socioeconomic factors reflect the driving forces of carbon storage spatial differentiation in the Western Sichuan Plateau to different degrees, and reduce the interference of human activities on the regional ecosystem, which is conducive to promoting the optimization of regional land ecosystem function.

#### Analysis of factor interaction detection based on geodetector

The interactive detection results of driving factors of carbon storage spatial differentiation are shown in Table 6.

**Table 6:** Interaction detection for spatial heterogeneity of carbon storage.

Factors	Rainfall	Temperature	NDVI	GDP	PD	HAI
Rainfall	0.0605					
Temperature	0.4501	0.3027				
NDVI	0.3484	0.4348	0.2561			
GDP	0.2388	0.3719	0.3627	0.0981		
PD	0.1809	0.3615	0.3103	0.1937	0.0877	
HAI	0.6055	0.6127	0.6338	0.5958	0.6024	0.5783

The results of interactive detection of driving factors for the carbon storage spatial differentiation in the Western Sichuan Plateau show (Table 6) that the interaction of any two driving factors is greater than that of a single driving factor, indicating that the effect of interaction between factors on carbon storage spatial differentiation is nonlinearly enhanced and interactively enhanced and the complex coupling between different factors jointly influences the effect of carbon storage spatial differentiation. There are 9 kinds of interactive enhancement, namely HAI ∩ Rainfall, HAI ∩ Temperature, HAI ∩ NDVI, HAI ∩ GDP, HAI ∩ Population density, Temperature ∩ NDVI, Temperature ∩ GDP, Temperature ∩ Population density and NDVI ∩ Population density. There are six kinds of linear enhancement, namely Rainfall ∩ Temperature, Rainfall ∩ NDVI, Rainfall ∩ GDP, Rainfall ∩ Population density, NDVI ∩ GDP and GDP ∩ Population density.

The interaction between HAI and the other five driving factors has an impact on carbon storage spatial differentiation of more than 50%. The interactive detection value q between HAI and NDVI was 0.6338, and the interaction had the greatest influence. The interaction influence between rainfall and temperature, temperature and NDVI were 0.4501 and 0.4348, respectively, indicating that the interaction had a great influence on carbon storage spatial differentiation. The interaction influence between rainfall and population density, GDP and population density is relatively small, only about 19%. From the interaction results, the interaction between HAI and NDVI and other natural factors is stronger than the interaction between internal social factors and internal natural factors.

## Discussion

The spatio-temporal evolution of ecosystem services is the result of complex coupling of natural factors and socioeconomic factors, but relevant studies lack to explore the driving mechanism of ecosystem services evolution from a spatial perspective, and the spatial direction in the regulation and optimization of regional ecosystem functions is ambiguous^32^. In this study, the modified InVEST model was used to estimate the carbon storage of the Western Sichuan Plateau, and the carbon storage of Aba Tibetan and Qiang Autonomous Prefecture was calculated according to the carbon storage value^48^. The results are only 4.2% different from the 711.3 billion yuan obtained by the Key Research and Development Program of Science and Technology Plan in Sichuan Province (2017SZY0007)^49^, indicating that this study has achieved a good effect. From 2020 to 2020, the overall trend of carbon storage in the Western Sichuan Plateau was “basically stable-rapid increase-slow decrease”, showing an overall trend of increase. According to relevant studies, the ecological service function of the Qinghai-Tibet Plateau increased steadily from 1995 to 2015, and the research results are consistent with the conclusion of this study^50,51^. Among them, the carbon storage increased significantly from 2005 to 2010, which is closely related to the implementation of the Natural Forest Protection Project in 2000^52^. Land use has a great impact on carbon storage^53,54^. After 2010, with the acceleration of urbanization, the increase of infrastructure construction and population in the study area, the intensity of land use transfer intensified, resulting in the reduction of carbon storage.

From the spatial cluster characteristics of carbon storage, the overall trend of the High-High Cluster and Low-Low Cluster influence of carbon storage gradually weakened in the past 20 years, indicating that the interference to the ecosystem in the study area has increased. According to the *Statistical Yearbook of Sichuan Province*, the population of the study area increased by 8% and the built-up land increased by 182.80 km^2^ from 2000 to 2020. The continuous expansion of human settlements and intensive grazing near the settlements in high mountain and plateau areas exacerbated grassland degradation^54^. However, the Western Sichuan Plateau has a low temperature and low accumulated temperature, and the recovery period of grassland and woodland is long after destruction^55^. Geological disasters also have a huge impact on the ecosystem^56^ According to statistics, over 12,000 geological disasters such as landslides and debris flows occurred in the study area in the past 20 years, and the areas with high incidence of geological disasters are mainly located in mountain and ravine areas, which is an important reason for the reduction of carbon density on both sides of roads and rivers.

The calculation results based on the Pearson correlation coefficient method show that among the impact factors of carbon storage changes in the Western Sichuan Plateau, HAI is the most influential factor and has the highest proportion in the significant positive correlation area; followed by GDP and NDVI, and other impact factors are less influential. From the perspective of spatial pattern of correlation, the correlation between different impact factors and carbon storage changes in different areas is quite different, which is related to the regional geological environment, topography, latitude, climate, land cover and soil texture^57,58^. The detection results of the carbon storage spatial differentiation in the study area show that HAI is the dominant factor, followed by temperature and NDVI change, and other factors have relatively little influence. The interaction detection results of driving factors show that the interaction of any two driving factors is greater than that of a single driving factor, indicating that the change of regional carbon storage is the result of the joint action of multiple factors, which is consistent with other relevant research results^59,60^. It shows that under the constraints of meteorological and geographical environment, the spatial pattern of carbon storage will have different effects due to the influence of human activities. The results show that under the constraints of meteorological and geographical environment, the spatial pattern of carbon storage will have different influences due to human activities. Therefore, in the process of ecological risk regulation and ecological environment protection, the characteristics of different driving factors should be taken into account and diversified regulation strategies should be adopted. For example, the protection and restoration of alpine wetland and river wetland, the protection of marsh grassland and the prevention and control of desertification should be emphasized in high mountain and plateau areas, while the protection of forest and biodiversity and the management of geological disasters should be emphasized in mountain and ravine areas.

From 2000 to 2020, the GDP in Western Sichuan Plateau had increased from 5.994 billion yuan to 82.2236 billion yuan, and the carbon stockage had grown from 1.2438 × 1010 t to 1.2455 × 1010 t, which indicated that ecological protection policies or regulatory frameworks including The Natural Forest Protection Project, Regional Ecological Construction and Environmental Protection Plan of The Qinghai-Tibet Plateau (2011–2030), Ecological Protection and Construction Plan for Tibetan Areas Of West Sichuan (2013–2020) had a significant effect on the improvement of regional ecological environment and the GDP increase.

## Conclusion

Taking the Western Sichuan Plateau as the research object, based on remote sensing, geographic information and socioeconomic data, the modified InVEST model is used to estimate the regional carbon storage in recent 20 years, and the geodetector is used to detect the carbon storage spatio-temporal differentiation in this study. The main conclusions are as following:

1. From 2000 to 2020, the carbon storage with an annual increase of 8.4 × 10^5^ t, the increasing area of carbon storage is mainly distributed in Litang County and Daofu County, and the decreasing area is mainly distributed along Ruoergai County and Minjiang River system in northeast China, while the other areas are mainly distributed in a sporadic and scattered manner. From the perspective of spatial cluster characteristics of carbon storage, High-High Cluster and Low-Low Cluster areas decreased by 1481.81 km^2^ and 311.11 km^2^ respectively, indicating that the spatial cluster characteristics of carbon storage are obvious in the Western Sichuan Plateau, but the overall effect is gradually weakening and the interference to the ecosystem is increasing.
2. The correlation analysis between carbon storage change and the impact factors shows that the carbon storage change in the last 20 years is most influenced by HAI, GDP and NDVI, which are the main factors leading to the increase or decrease of carbon storage during the period. Geographical detection analysis shows that HAI is the dominant factor in the carbon storage spatial differentiation, followed by temperature and NDVI, while other factors are relatively small. After the interaction between factors, the influence on carbon storage spatial differentiation is nonlinearly enhanced and interactively enhanced, indicating that carbon storage change in the Western Sichuan Plateau is the result of the combined influence of natural factors and socioeconomic factors, but the socioeconomic factors play a stronger role.
3. Under the current ecological protection measures, the ecosystem service functions of the western Sichuan plateau are developing in a positive way. However, what needs to be paid attention to is the obvious reduction of carbon storage in the high mountain and plateau areas represented by Ruoergai County and mountain and ravine areas represented by Wenchuan County and Danba County. Therefore, it is necessary to strictly implement the ecological protection red line, rationally control the development scale of built-up land, and adopt differentiated ecological regulation models and strategies to improve the stability of the ecosystem, enhance carbon storage capacity.
4. LUCC has an impact on the emission and absorption processes between terrestrial ecosystems and the atmosphere, and is the most closely related to human activities and ecological environment. The research plays a positive role in exploring the development of land use carbon emission standards, providing a reference for decision making on the optimization of national land spatial pattern oriented to carbon peaking and carbon neutrality, and making new contributions to the IPBES global ecosystem service assessment as well as the prevention of global warming proposed by IPCC AR6.

## Data Availability

The data that support the findings of this study are available from the corresponding author upon reasonable request.
